# Supplementary material for: Parallelized multidimensional analytic framework applied to mammary epithelial cells uncovers regulatory principles in EMT
Source: Nat Commun. 2023 Feb 8;14:688. doi: 10.1038/s41467-023-36122-x (PMC9908882; doi:10.1038/s41467-023-36122-x)
Supplement: Supplementary file 1 — Supplementary Information [file 41467_2023_36122_MOESM1_ESM.pdf]

Supplementary Figures

Parallelized multidimensional analytic framework applied to mammary epithelial cells uncovers regulatory principles in EMT

Indranil Paul[1], Dante Bolzan[2], Ahmed Youssef[3], Keith A. Gagnon[4], Heather Hook[5,6], Gopal Karemore[7], Michael U.J. Oliphant[8], Weiwei Lin[1], Qian Liu[9], Sadhna Phanse[1], Carl White[1], Dzmitry Padhorny[10,11], Sergei Kotelnikov[10,11], Christopher S. Chen[4,12], Pingzhao Hu[13], Gerald V. Denis[14], Dima Kozakov[10,11], Brian Raught[15], Trevor Siggers[5,6], Stefan Wuchty[2†], Senthil K. Muthuswamy[16†], Andrew Emili[1,17†*]

Supplementary Figure 1

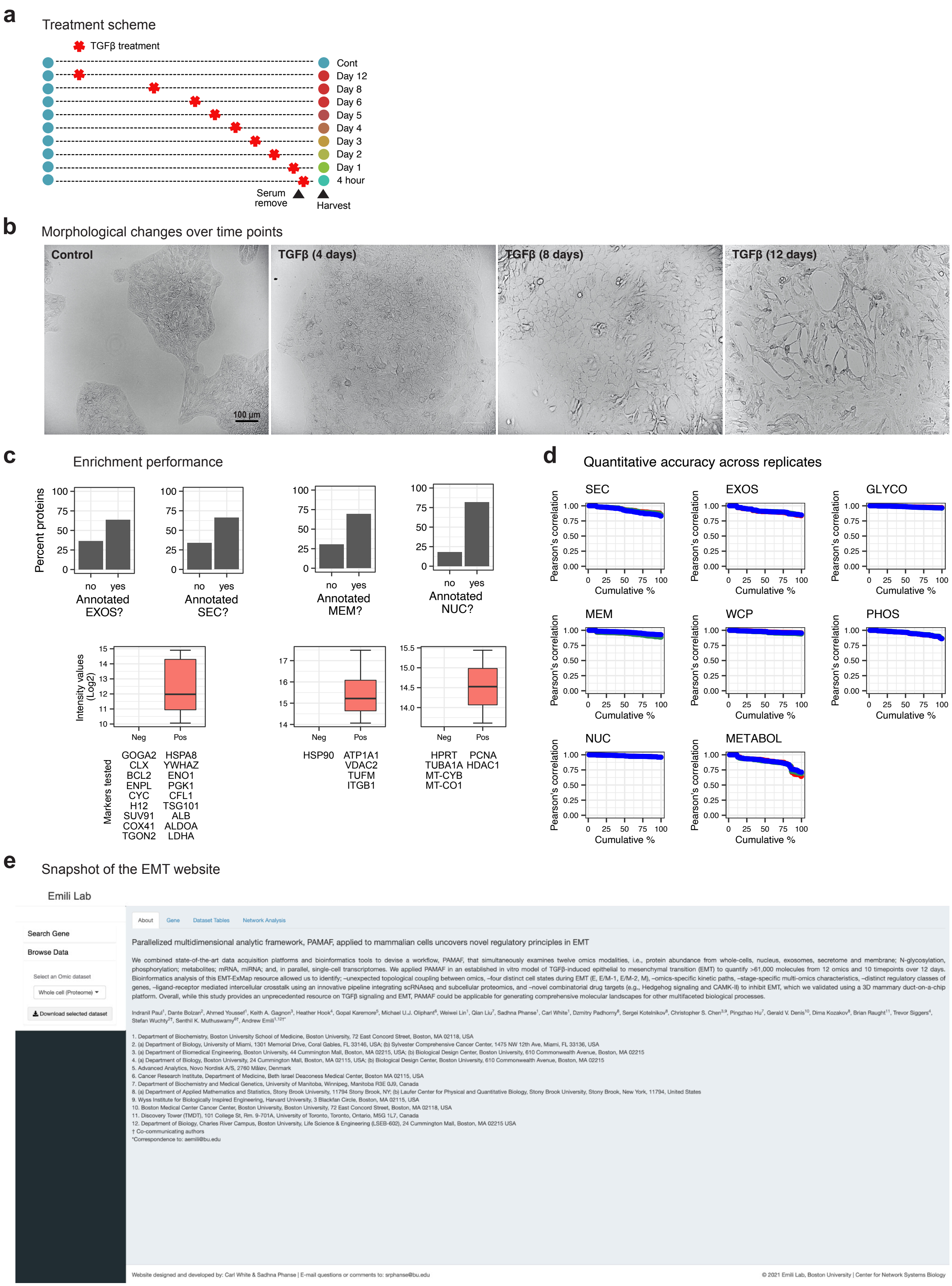

Supplementary Fig. 1. Experimental design, quality analysis and companion website

a) Time course TGFβ treatments were staggered such that all plates were harvested at the same time. Cells were serum starved for 16 hours before harvesting, to facilitate extraction of EXOS and SEC layers and to reduce background in PHOS.

b) Phase-contrast images of MCF10A cells at different time points after TGFβ treatment. Scale bar is shown on the first image. Representative images from three independent experiments are shown.

c) Upper panel – bar plots showing % of quantified proteins with the respective annotation (x axis label) in the 'Cellular Component' category (Source Data file – Supp Fig 1c-1). Lower panel – box plots showing intensity values (log2) of proteins (=markers) commonly used to assess sub-cellular fractionation purity (Source Data file – Supp Fig 1c-2). The centerline of box plot denotes the median; lower and upper bounds indicate 1st and 3rd quartiles, respectively; whiskers reach the maximum and minimum point within the 1.5x interquartile range. Subcellular enrichments were performed using previously established MS-compatible protocols (see Methods), yielding high purity as determined through keyword matching against the GO cellular compartment annotation database.

d) Cumulative distribution (%) of PCCs across the time points (Source Data file – Supp Fig 1d). The overlap between the three biological replicates, shown as three colors, indicates excellent reproducibility.

e) A snapshot of the companion EMT website.

Supplementary Figure 2

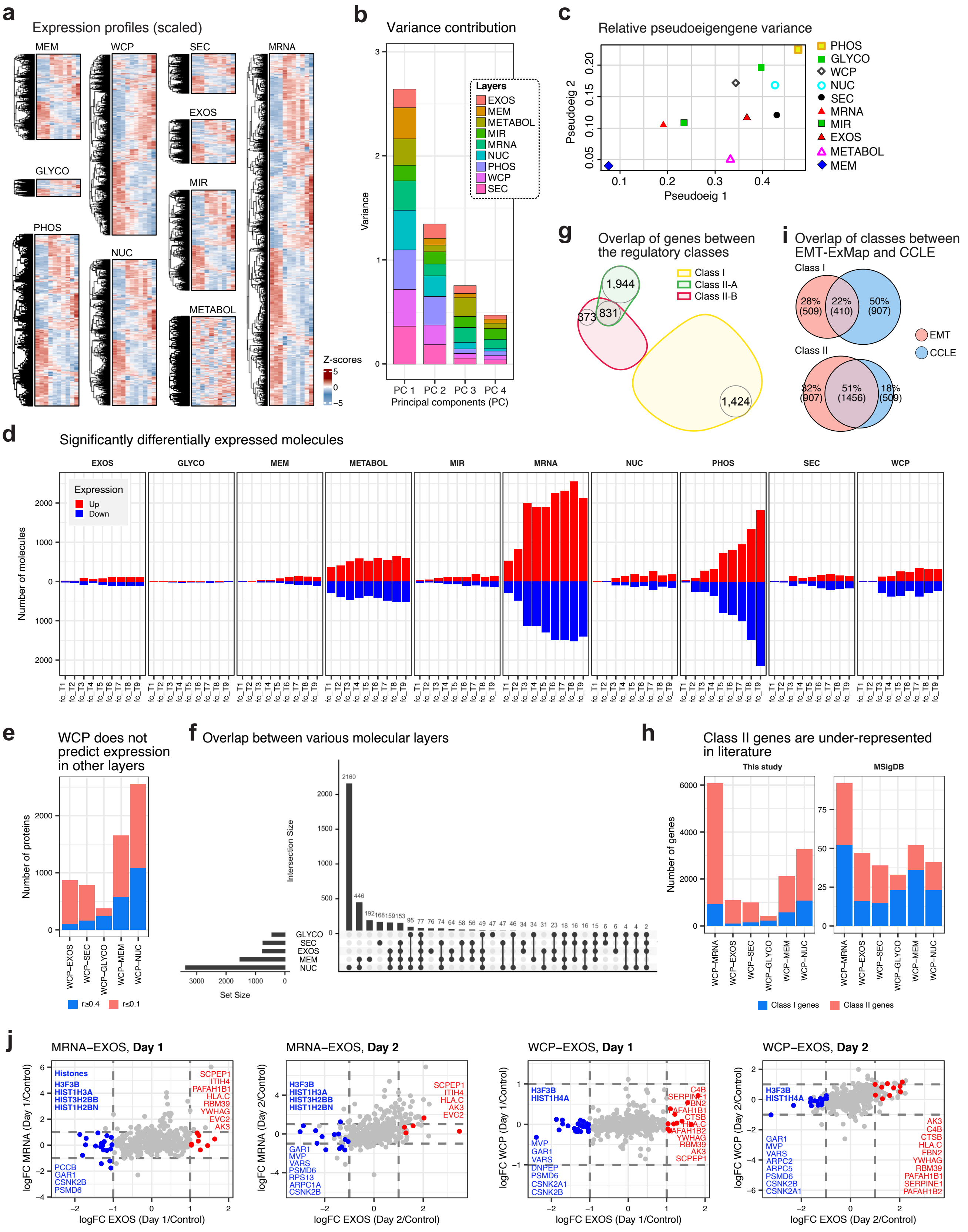

Supplementary Fig. 2. Extensive alterations and regulatory autonomy between layers

a) Heatmaps of Z-scored molecular expressions of each layer quantified in EMT-ExMap over the time points. Mean expression across the three replicates was used.
b) Variance contributed by each layer over 4 principal components. The omicade4 R package was used.
c) This plot shows a 2-dimensional representation of pseudo-eigenvalues, indicating how much variance is contributed by each dataset in relation to the eigenvalue space of all datasets. The omicade4 R package was used.
d) Barplot showing the number of regulated molecules at each time point for each layer.
e) Barplot showing number of proteins in various proteomic layers with correlated (PCC ≥ 0.4) and un- or anti-correlated (PCC ≤ 0.1) expression with respect to WCP.
f) The plot shows number of common genes (intersection size, y axis) between the layers as indicated. Only regulated genes in each layer (=set size) were considered for the analysis.
g) The Venn diagram shows overlaps between genes of Class I, Class II-A and Class II-B (Source Data file – Supp Fig 2g).
h) Barplot showing the distribution of Class I and II genes in EMT-ExMap (this study) and MSigDB hallmarks.
i) Comparison of gene classifications computed from the CCLE database to those derived from our EMT-ExMap for the set of 2,297 overlapping genes. Since the CCLE database has multiple tissues, each gene can have multiple different classifications in the CCLE database, i.e., one per tissue. To simplify, we used a majority voting approach to assign each gene in the CCLE database to one category based on its most common classification across the 14 tissues. We found that 56.90% of genes had the same classification in both datasets (Source Data file – Supp Fig 2i).
j) Scatterplots depict the overlap of regulated genes in EXOS and either MRNA or WCP after day 1 and day 2 of TGFβ treatment.

Supplementary Figure 3

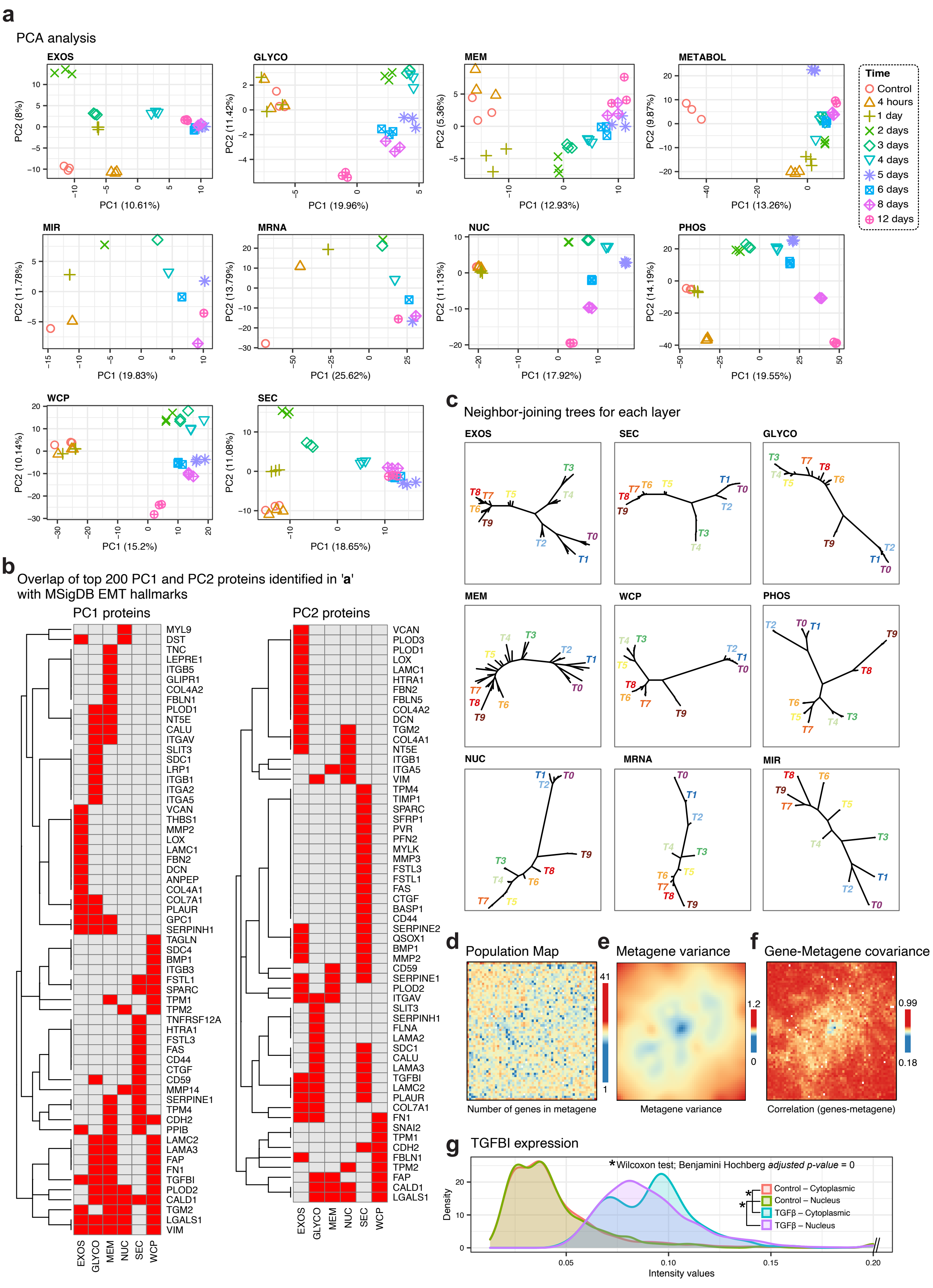

Supplementary Fig. 3. Topological relation and kinetics of molecular layers

a) Principal component analysis (PCA) of the regulatory layers. Time points are shown with different shapes and colors. Points with similar shape/color indicate biological replicates.
b) The heatmap shows the overlap of genes associated with PC1 and PC2 from the PCA analysis in panel 'a' above with MSigDB EMT hallmarks. It shows that both PC1 and PC2 contains important information with respect to EMT progression.
c) Phylogenetic neighbor-joining trees constructed using individual layers. Although the overall evolutionary dynamics of EMT is comparable the exact kinetics are quite different for each layer, indicating that each layer evolved distinctly during EMT.
d) The population map displays the number of features in each metagene which ranges between 1 to 41 as shown in the scale bar.
e) The plot summarizes co-variance structure of datasets at the metagene level. The scale bar shows that the blue spot in the center contains invariant metagenes. The more variant and higher populated metagenes are located along the borders that are suited for classification analysis between the samples.
f) The plot shows correlation scores in absolute scale of expression patterns of individual features contained within metagenes. The map illustrates the concerted changes of features in each of the metagene clusters, showing that the Euclidian distance-based SOM algorithm provides robust clustering of correlated expression profiles together in different regions of the SOM. The scores range between 0.18 and 0.99 as shown in the scale bar.
g) Distribution of intensity values of TGFBI staining (Source Data file – Supp Fig 3g). The *adj. p-value* shows the significance of difference between the indicated groups as evaluated using unpaired two-sided Wilcoxon tests (n>1,500 cells in each group), with Benjamini-Hochberg correction for multiple testing. Representative results are displayed from two independent experiments.

Supplementary Figure 4

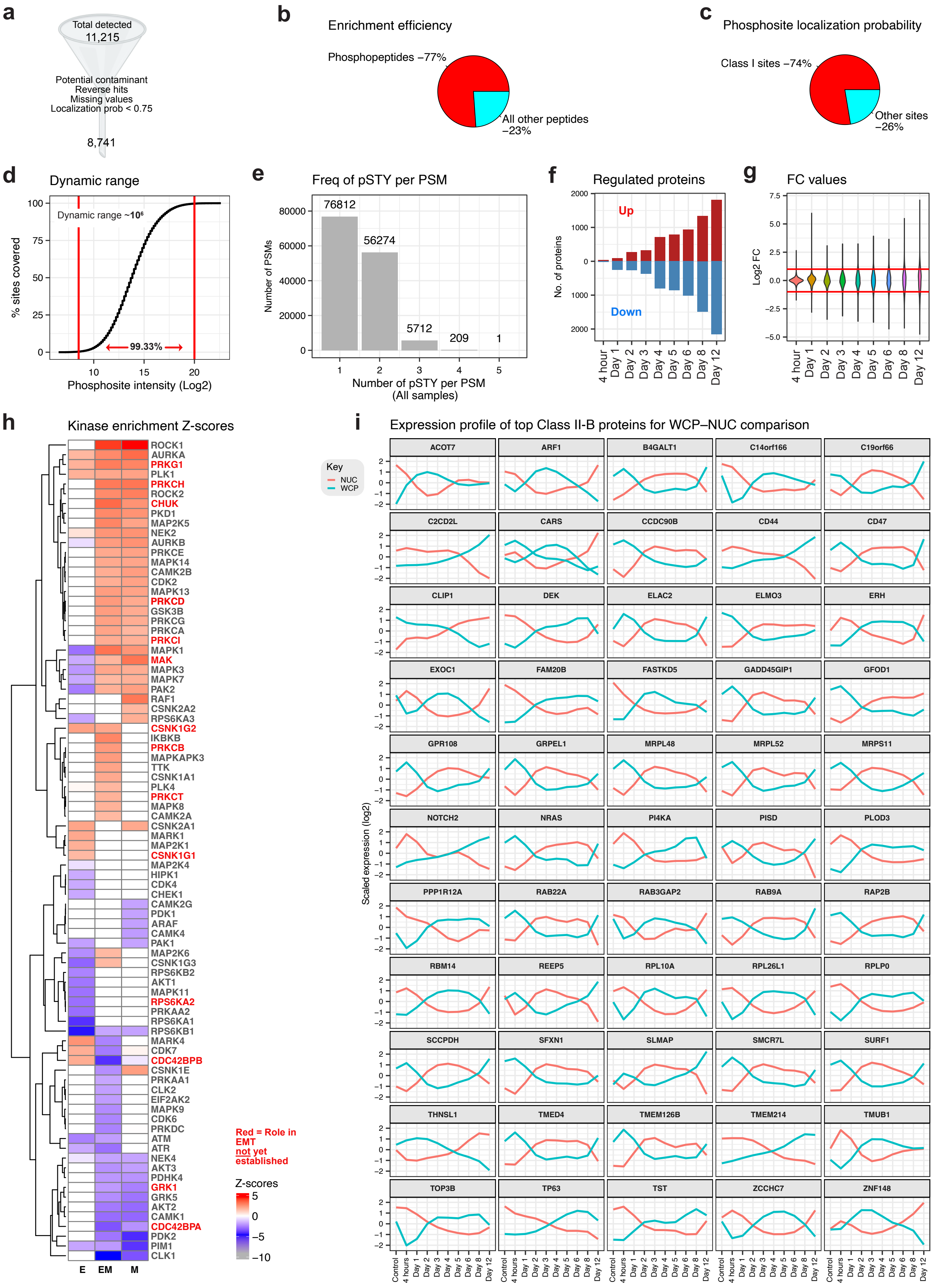

Supplementary Fig. 4. Phosphoproteomics analysis

a) An outline of the QC pipeline used for processing of PHOS data. Parts of this panel were created using BioRender.
b) About 77% of detected peptides across all three replicates were phosphopeptides, showing high enrichment efficiency (Source Data file – Supp Fig 4b).
c) About 74% of detected p-sites across all three replicates were reliably localized (i.e., localization probability ≥ 75% = Class I) by MaxQuant (Source Data file – Supp Fig 4c).
d) Quantifications of p-sites were achieved with a dynamic range of 10^6 orders of magnitude (Source Data file – Supp Fig 4d).
e) Proportions of number of phosphate moieties in each detected p-site across all three replicates (Source Data file – Supp Fig 4e).
f) Number of phosphoproteins regulated over the time course.
g) Magnitudes of log2FC values for p-sites over the time course.
h) The heatmap shows the Z-scores from kinase enrichment analysis using KSEAapp, averaged over the three stages of EMT (Source Data file – Supp Fig 4h).
i) Expression profiles of Class II-B proteins with PCC ≤ –0.8 between WCP and NUC, indicating opposite expression and suggesting re-localization into, or out of, NUC during EMT.

Supplementary Figure 5

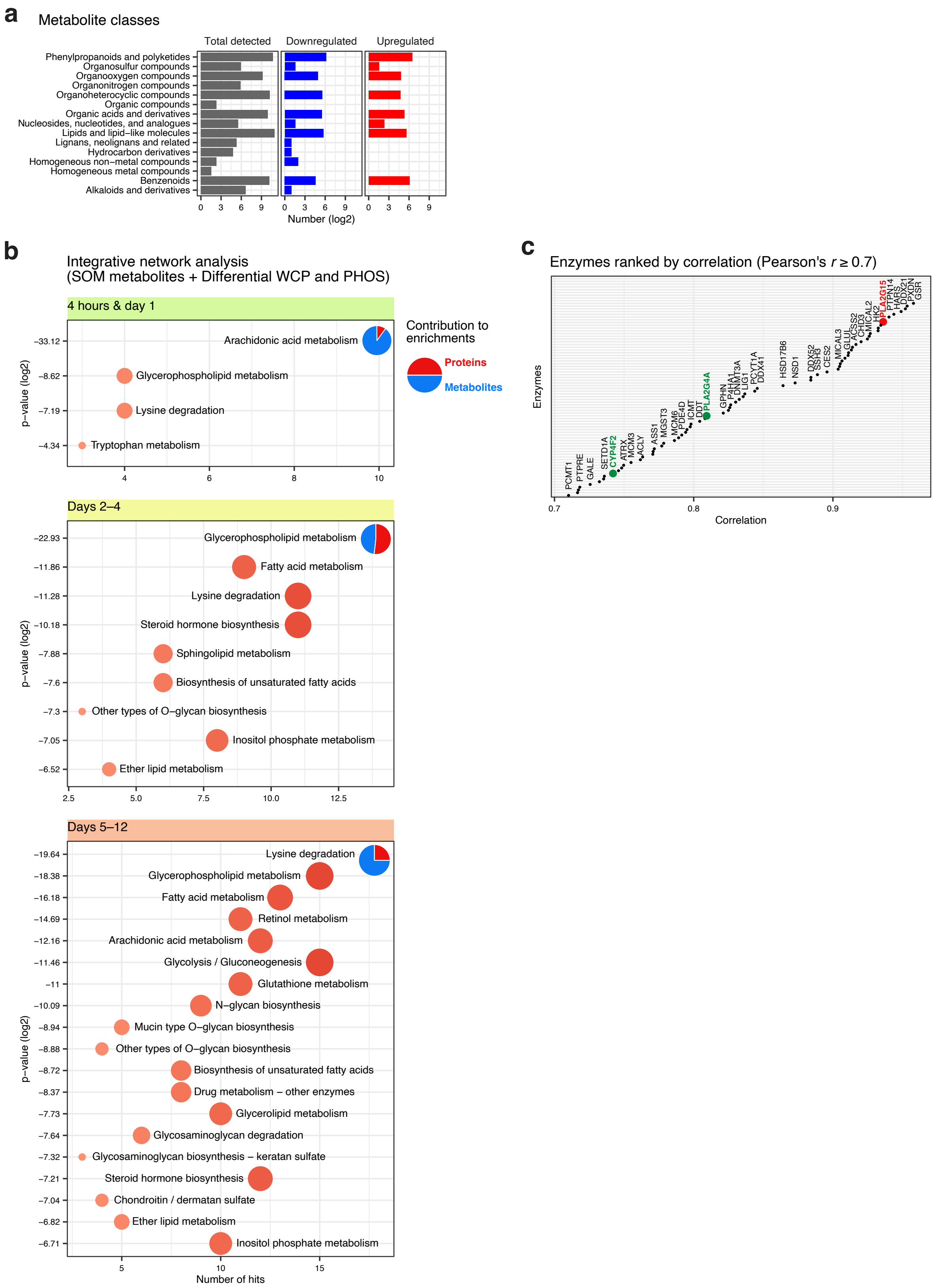

Supplementary Fig. 5. Analysis of metabolomics data

a) Barplot showing the ‘class’ distribution of quantified metabolite features in HMBD. Also shown are the relative proportions of regulated features of each class (*p-value*≤0.01, |log2FC|≥1) determined using maSigPro as described previously (Source Data file – Supp Fig 5a).

b) Top 10% of metabolite features ranked by SOM were grouped based on clusters in ‘Fig. 5a’ and used for ‘Joint network analyses’ with regulated molecules in WCP and PHOS using MetaboAnalyst (https://www.metaboanalyst.ca). The *p-values* are estimated with a weighted Z-test as implemented in MetaboAnalyst.

c) KEGG annotated metabolic enzymes were ranked according to their PCC with metabolites of the AAM pathway quantified in EMT-ExMap (Source Data file – Fig 5b).

Supplementary Figure 6

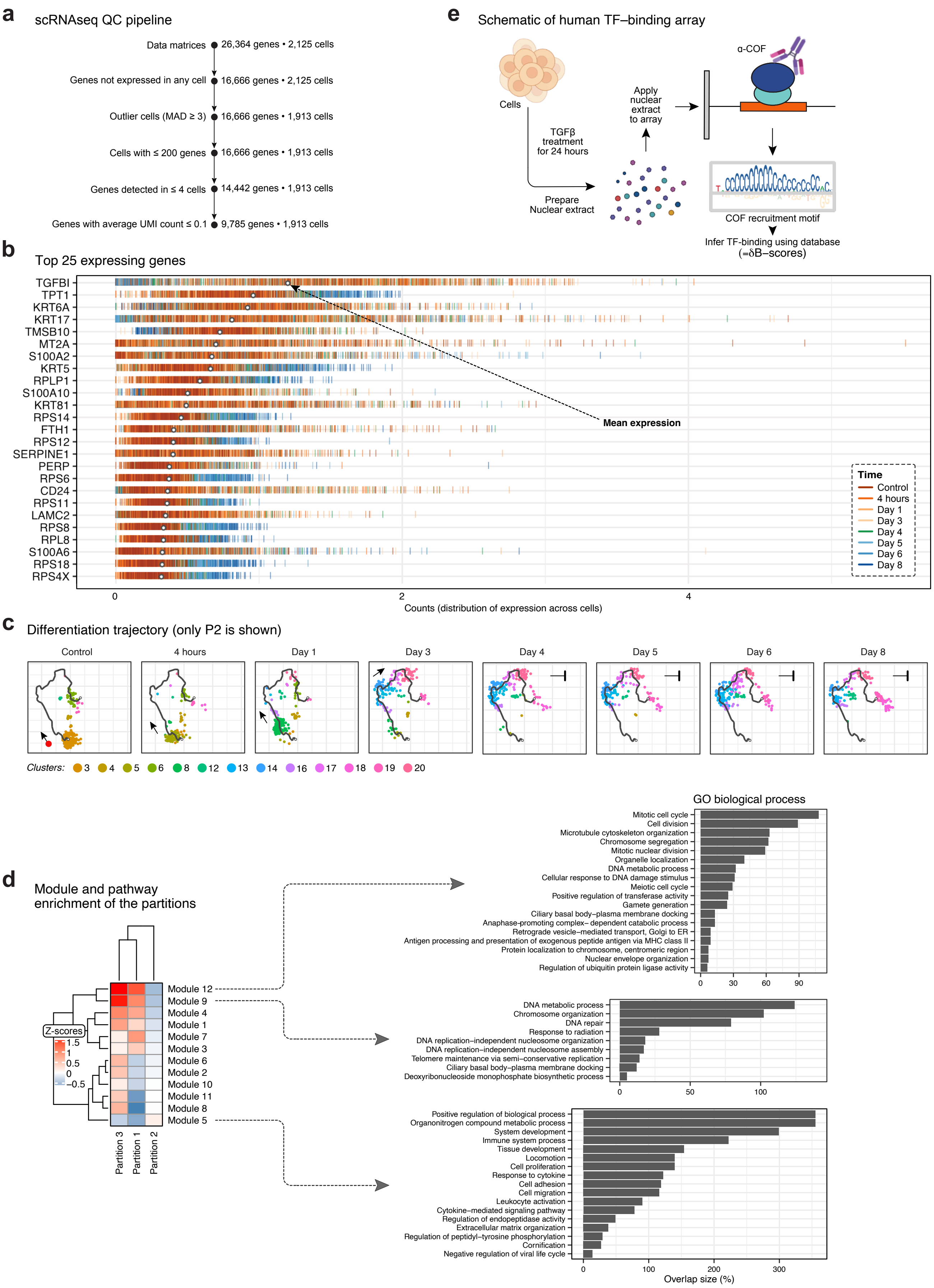

Supplementary Fig. 6. Analysis of scRNAseq data

a) An outline of the QC pipeline employed for scRNAseq data processing.
b) The plot shows the top 25 most expressed genes in the scRNAseq dataset across all samples. Each row corresponds to a gene and each colored bar points to its expression in single cells in various time points.
c) Developmental trajectories of MCF10A cells in response to TGFβ, inferred by Monocle3. Clusters are indicated by colors. Cells evolved swiftly in response to TGFβ for up to Day 3 (shown as →), after which the clusters 'stabilized' (shown as —|).
d) Heatmap showing aggregate expression of groups of genes (=modules) with similar expression pattern across the partitions inferred by Monocle3. Modules 9 and 12 were highly expressed in P1 and P3 and were mostly enriched for 'cell cycle' related GO annotations (Source Data file – Supp Fig 6d). Enrichment analysis was performed using the gProfileR R package. The *p-values* estimated using hypergeometric testing were adjusted for multiple comparisons using the Benjamini-Hochberg method.
e) Schematics of the human TF-binding array workflow. See Methods for details. Parts of this panel were created using BioRender.

Supplementary Figure 7

a EMT network: A hierarchical causal signaling network

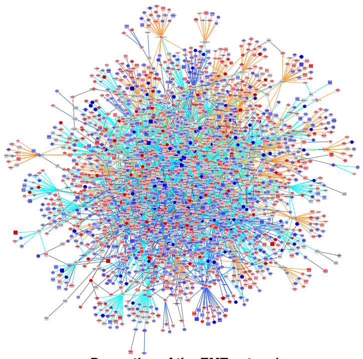

Properties of the EMT network
Total number of nodes: 2,217
Total number of edges: 3,255
Average number of neighbor: 2.970

b List of all controllers identified in this study (alphabetical)

ABL1	CHEK1	IL1B	PDX1	SPRY4
AKT1	CREBBP	ILK	PLCG1	SRC
AKT2	CTNNB1	IRF3	PPP1CA	SREBF1
APC	CYP11B2	JUN	PPP1CC	SRF
APOB	DAXX	KRT1	PPP2CA	SRPK2
APP	DVL1	MAP3K5	PPP2CB	STAT1
ARVCF	E2F1	MAP3K7	PPP2R2D	STAT2
ATF2	EBF1	MAPK1	PPP3CA	STAT3
ATF4	EGFR	MAPK14	PRKAA1	STK4
ATM	EGR1	MAPK3	PRKACA	TAL1
ATR	ELL	MAPK8	PRKCA	TBP
AURKA	EP300	MDC1	PRKDC	TCF4
AURKB	ERBB2	MDM2	PTEN	TGFB1
BCL10	ESR1	MEF2A	PTK2	TGFBR1
BCL2	ESRRA	MMP14	PTPRS	TGFBR2
BCL2L1	ETS2	MMP2	RAC1	TNFSF10
BCL3	FBN1	MTA1	RAD21	TP53
BRCA1	FIG4	MTOR	RAF1	TRAF2
BTF3	FOXO1	MYC	RBPJ	TRAF6
CASP3	FYN	MYOD1	REST	TRIM28
CASP7	GABARAP	NBR1	RHEB	TTL
CASP9	GATA2	NCOR2	RHOA	UBE2N
CBL	GNB1	NFE2L2	RNF4	VCP
CDC25A	HBB	NFKB1	RPS6KB1	VRK1
CDH4	HDAC1	NOTCH1	RUNX1	WT1
CDK1	HIF1A	NPM1	SHC1	YAP1
CDK14	HSP90AA1	NR3C1	SIN3A	ZEB1
CDK2	HTRA2	PAK1	SMAD3	ZNRF3
CDON	IL1B	PAX3	SMO	
CHD4		PBX3	SPI1	

c KM plot for altered expression of each signature set

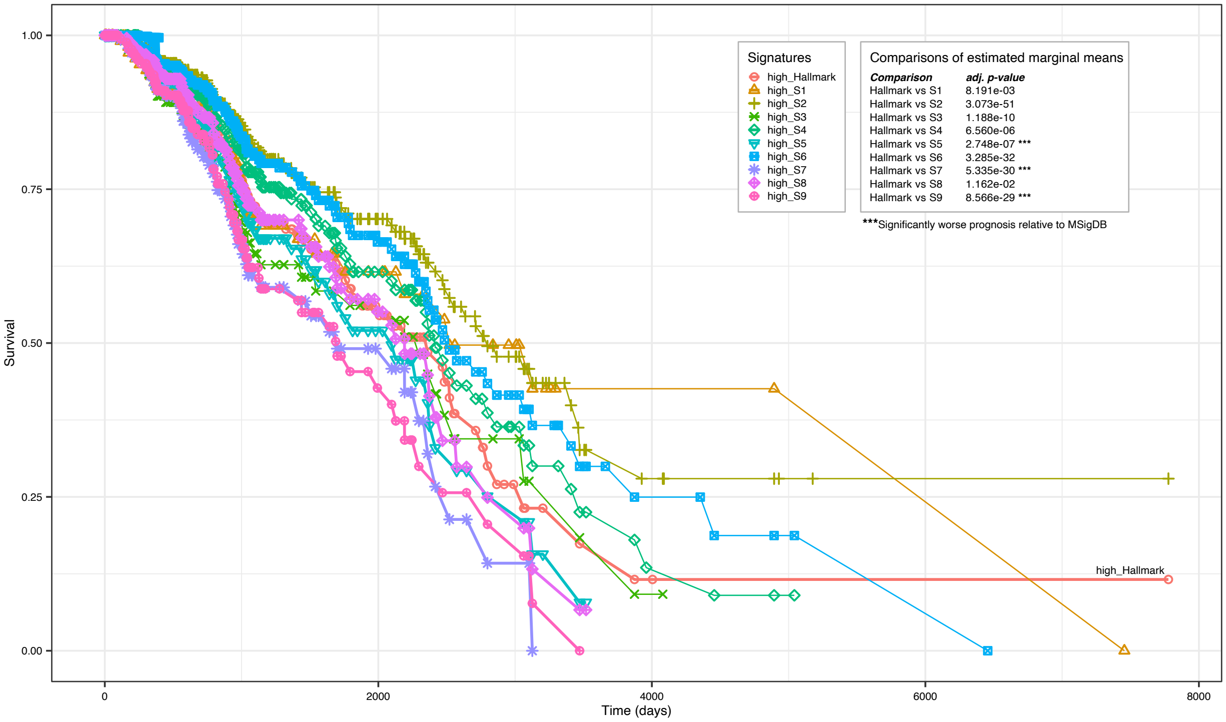

d Workflow for morphometric analysis of inhibitor screen

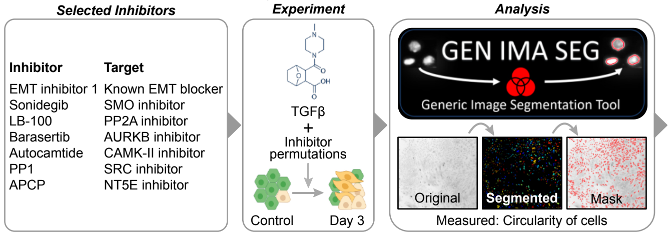

e Exposed combinatorial vulnerabilities

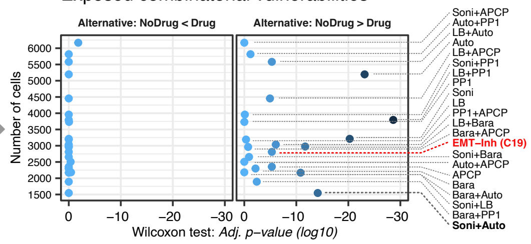

Supplementary Fig. 7. Integrative EMT network and drug screening

a) Integrative causal EMT network constructed using differential molecules in EMT-ExMap.
b) List of controllers identified using controllability analysis of the EMT network (see Methods).
c) To evaluate if the survival curves for altered expression of MSigDB hallmarks and our S1 to S9 signatures are statistically different, we compared estimated marginal means of the curves of each signature using paired one-way ANCOVA. Adjusted *p-values* were computed using 'emmeans_test' with Benjamini-Hochberg correction for multiple testing, as implemented in rstatix R package.
d) Workflow for morphometric screening of drug combinations. MCF10A cells grown in 24-well plates were treated with drugs either individually or in combination along with TGFβ for 3 days. Brightfield phase-contrast images were captured using the Celigo Imaging Cytometer in a semi-high-throughput fashion and were analyzed using the GenImaSeg tool. We computed 'eccentricity' (i.e., asymmetric elongation) to compare the cellular phenotypes across conditions.
e) All cells were treated with TGFβ. For each drug or drug combination, the ratio of 'eccentricity' values between NoDrug and Drug treated cells were calculated to evaluate the effect of drug treatment in specifically inhibiting TGFβ-induced EMT. The significance of difference between the NoDrug and Drug conditions was then tested using unpaired one-sided Wilcoxon tests with alternative hypothesis, as shown. The NoDrug condition was used as the reference (Source Data file – Supp Fig 7e). The *y* axis shows the number of cells in each group and the *x* axis shows the Benjamini-Hochberg adjusted *p-values* (log10 transformed). The drug 'EMT inhibitor (C19)' is known to inhibit EMT and is used as a positive reference. We chose Sonidegib + Autocamtide (highlighted in red) for further validation.
