# Supplementary material for: Parallelized multidimensional analytic framework applied to mammary epithelial cells uncovers regulatory principles in EMT
Source: Nat Commun. 2023 Feb 8;14:688. doi: 10.1038/s41467-023-36122-x (PMC9908882; doi:10.1038/s41467-023-36122-x)
Supplement: Supplementary file 3 — Description of Additional Supplementary Files [file 41467_2023_36122_MOESM3_ESM.pdf]

Supplementary Data Files Legends

Parallelized multidimensional analytic framework applied to mammary epithelial cells uncovers regulatory principles in EMT

Indranil Paul[1], Dante Bolzan[2], Ahmed Youssef[3], Keith A. Gagnon[4], Heather Hook[5,6], Gopal Karemore[7], Michael U.J. Oliphant[8], Weiwei Lin[1], Qian Liu[9], Sadhna Phanse[1], Carl White[1], Dzmitry Padhorny[10,11], Sergei Kotelnikov[10,11], Christopher S. Chen[4,12], Pingzhao Hu[13], Gerald V. Denis[14], Dima Kozakov[10,11], Brian Raught[15], Trevor Siggers[5,6], Stefan Wuchty[2†], Senthil K. Muthuswamy[16†], Andrew Emili[1,17†]*

Supplementary Data file 1. Expression datasets of omic layers in EMT-ExMap

This table provides the quantification values of all features across the time points and replicates retained after the quality control (QC) steps (see Methods for details)

Supplementary Data file 2. Differential analysis of omic layers in EMT-ExMap

This table provides the differential expression values (as log2FC) of all significant features across the time points (see Methods for details)

Supplementary Data file 3. SOM analysis & pathway enrichment

This table provides the SOM molecular portraits as discussed in Fig. 2 in the main text. Also provides the results of 'pathfindR' analysis (see Methods) of the SOM portraits with features with *adj.p-value* ≤ 0.05 and logFC ≥ 0.6

Supplementary Data file 4. CC correlation of Class I and class II proteins

This table provides the list of Class I & Class II genes as described in this study. The values represent Pearson's correlation coefficient of genes (rows) between the indicated omic layers (column names)

Supplementary Data file 5. scRNAseq subtype & GOBP analysis

Provides the list of enriched TFs (using SCENIC analysis) in each of the major cell subtypes defined in this study. The 'GOBP_modules' provides the enrichment results of 'gene ontology – biological process' terms of genes associated with the modules identified from Monocle3 analysis of the scRNAseq dataset

Supplementary Data file 6. GenImaSeg output

This table provides the output of the GenImaSeg analysis in Supplementary Fig. 7

Supplementary Data file 7. Softwares and algorithms

This table enlists the various softwares and algorithms used in this study, along with a brief description of how the tools were specifically used in this study. Direct web links of their internet locations are also provided.
